# Students’ readiness for and perception of Interprofessional learning: a cross-sectional study

**DOI:** 10.1186/s12909-020-02325-9

**Published:** 2020-10-29

**Authors:** Arwa Alruwaili, Noora Mumenah, Nesrin Alharthy, Fatmah Othman

**Affiliations:** 1 Department of Respiratory Therapy, College of Applied Medical Sciences, King Saud bin Abdul Aziz University for Heath Sciences, P.O. Box 3660, Riyadh, 11481 Saudi Arabia; 2 King Abdullah International Medical Research Centre, Riyadh, Saudi Arabia; 3 Pediatric Emergency Medicine, King Abdulaziz Medical City, National Guard-Health Affairs, Riyadh, Saudi Arabia; 4 Research Unit, College of Applied Medical Sciences, King Saud bin Abdul Aziz University for Heath Sciences, Riyadh, Saudi Arabia

**Keywords:** Interprofessional learning, Interprofessional, Undergraduate students, Applied medical sciences, Healthcare professional, Readiness and perception

## Abstract

**Background:**

Several studies reported that Inter-professional Education (IPE) plays a major role in creating an effective collaborating environment in healthcare settings to achieve high-quality patient care. This study measured the College of Applied Medical Sciences (CAMS) students’ readiness for and perceptions of IPE.

**Methods:**

A cross-sectional study was conducted with 232 undergraduate students in Riyadh, using a stratified random sampling method. All the undergraduate students of CAMS were included. Two previously validated questionnaires, the Interdisciplinary Education Perception Scale (IEPS) and the Readiness for Interprofessional Learning Scale (RIPLS) were used in the study.

**Results:**

The mean score for the RIPLS was 86.8. The Tukey post-hoc test score was significantly higher comparing the Occupational Therapy and the Respiratory Therapy programs. There was no difference between the overall RIPLS and subscales between male and female students as well as senior and junior students. For the IEPS, the mean score was 77.7. The Tukey post-hoc test score was significantly higher in the Occupational Therapy and Respiratory Therapy programs.

**Conclusion:**

The current study indicated that the Applied Medical Sciences’ students demonstrated readiness for IPE as an important element in creating collaborative teamwork in their programs. The early incorporation of IPE in the pre-professional years will enhance collaboration in management and patient care.

**Supplementary Information:**

The online version contains supplementary material available at 10.1186/s12909-020-02325-9.

## Background

Interprofessional Education (IPE) is a concept focusing on the development of good communication and a teamwork environment for all healthcare providers to achieve high-quality patient care [1, 2]. The Institute of Medicine (IOM) reported that healthcare providers have to be able to work within a multi-disciplinary team to deliver high-quality patient care [3], and the complexity of the system mandates the integration of strategies to maintain collaborative teamwork. Several studies stated that IPE plays a major role in creating an effective collaborating environment in a healthcare setting [4, 5], which resulted in a strong recommendation to implement IPE as an integral part in the curriculum of undergraduate medical and health related professions [1]. Several institutions have embedded IPE standards in their medical curriculum.

There are many arguments in literature regarding the effectiveness of exposing undergraduate students to IPE to promote interdisciplinary collaboration and teamwork. Current literature provide positive evidence of improvement in the professional identity and attitudes of undergraduate students to teamwork after embedding IPE in their curriculum [2, 4]. Literature indicates that students from Medical, Nursing, Physical Therapy, and Pharmacy have a positive attitude to working collaboratively [5–9]. The outcome of embedding an IPE module in the curriculum enhanced the acquisition of knowledge and skills in resolving complex issues [4].

The early interaction of students from different health disciplines facilitate the acquisition of the required skills to work effectively in a healthcare setting [2, 4]. Positive attitudes towards IPE increase the likelihood of a favorable outcome. Many instruments have been used to assess the perceptions of the students about IPE. However, the Interdisciplinary Education Perception Scale (IEPS), and the Interprofessional Attitudes Questionnaire, and the Readiness for Interprofessional Learning Scale (RIPLS), are the two instruments most frequently used [10, 11].

There are limited literatures related to the perception of Applied Medical Sciences students regarding IPE. The diversity of the professions of the Applied Medical Sciences could possibly influence the students’ attitudes and interactions. The majority of the studies examined the readiness and perception of IPL, with few studies investigating the topic in Applied Medical Science students. Applied Medical Science graduates play an important role in creating effective collaboration in healthcare settings to achieve high-quality patient care. The aim of this study was to assess the perceptions of and readiness for IPE of the undergraduate students in the College of Applied Medical Sciences (CAMS) within the different specialties. The undergraduate students at CAMS enroll in formal research methodology courses as a combined group, to foster teamwork skills and to support IPE. Evaluating the students’ perceptions of IPE will provide in-depth insight of their willingness for future interprofessional collaboration.

## Methods

### Study setting and population

We conducted a cross-sectional study with the undergraduate students in CAMS in King Saud bin Abdulaziz University for Health Sciences, Riyadh, from January 2019 to March 2019. All the CAMS undergraduates were included except for the Clinical Nutritional program, as the program had only female students. Students from the following programs were included: Respiratory Therapy program, Emergency Medical Services program, Occupational Therapy program, Radiology Science program, Clinical Laboratory Studies program, Anesthesia Technology program and the Cardiovascular Technology program. The curriculum of the programs consists of two phases: a Pre-Professional and a Professional phase. In the Pre-Professional phase, the students attend 2 years in the College of Science and Health Professions where they receive basic science courses, English language courses, and introductory courses related to their specialty. In the Professional phase, the students enroll for 2 years in CAMS, followed by 1 year of internship. Students in their third year of college are considered juniors and, in the fourth year, as seniors.

A stratified random sampling method was used to choose students from each program proportionally. The estimated sample size was calculated as 232 students using a total population size of 588, 5% margin of error, and 95% confidence level. We obtained a list from Student Affairs at CAMS, and we distributed the questionnaire accordingly. Informed consent was obtained from each student prior completing the questionnaire.

### Study instrument

Two self-administered validated questionnaires were used to assess the students’ readiness for IPE. We used two previously validated questionnaires, the Interdisciplinary Education Perception Scale (IEPS) and the Readiness for Interprofessional Learning Scale (RIPLS) [10, 11]. The two tools were validated and published in English and are licensed for public use. The research team distributed the self-administered questionnaires to the participants after obtaining informed consent from each participant. Each questionnaire was divided into two sections. The first section collected demographic information including the year of study, gender, and specialty. The second section contained the questionnaires. The RIPLS tool consists of 19 items in three subscales: teamwork and collaboration (items 1–9), professional identity (items 10–16), and roles and responsibility (items 17–19). The scoring system is a 5-point Likert-scale (1 = strongly disagree, 5 = strongly agree). The total score of this scale ranges from 19 to 95 with higher scores indicating greater readiness for IPE. The IEPS tool consists of 18-items. This instrument is scored with a 6-point Likert-scale (1 = strongly disagree, 6 = strongly agree). This scale involves four subscales: competence and autonomy (items 1, 3, 4, 5, 7, 9, 10 and 13), perceived need for cooperation (items 6 and 8), perception for actual cooperation (items 2, 14, 15, 16 and 17) and understanding others’ value (items 11, 12 and 18). The total score for this scale ranges from 18 to 108.

The study was approved by the Institutional Review Board IRB at King Abdullah International Medical Research Center, Riyadh (Protocol number RYD-19-419812-5854).

### Data analysis

Descriptive statistics were used for the demographic variables. The mean scores of the RIPLS and IEPS were compared between the programs. An one-way analysis of variance, ANOVA, was used to compare the mean scores for the different programs. Tukey’s post hoc test was used to analyze significant differences and the results were considered statistically significant if the *p*-value was < 0.05. All analyses were done using STATA version 12 (StataCorp. 2011. Stata Statistical Software: Release 12. College Station, TX: StataCorp LP).

## Results

### Demographic characteristics of the sample

In total, 233 undergraduate students completed both questionnaires. Just more than half of the sample (55%) was male and the participants were equally distributed in terms of level of study with 51% senior and 49% junior students. The proportions of the students from each program were 20% from the Respiratory Therapy program, 18% from the Emergency Medical Services program, 18% from the Occupational Therapy program, 12% from the Radiologic Science program, 6% from Clinical Laboratory Studies program, 14% from Anesthesia Technology program and 10% from the Cardiovascular Technology program.

### The readiness for Interprofessional learning scale (RIPLS)

**Fig. 1:**
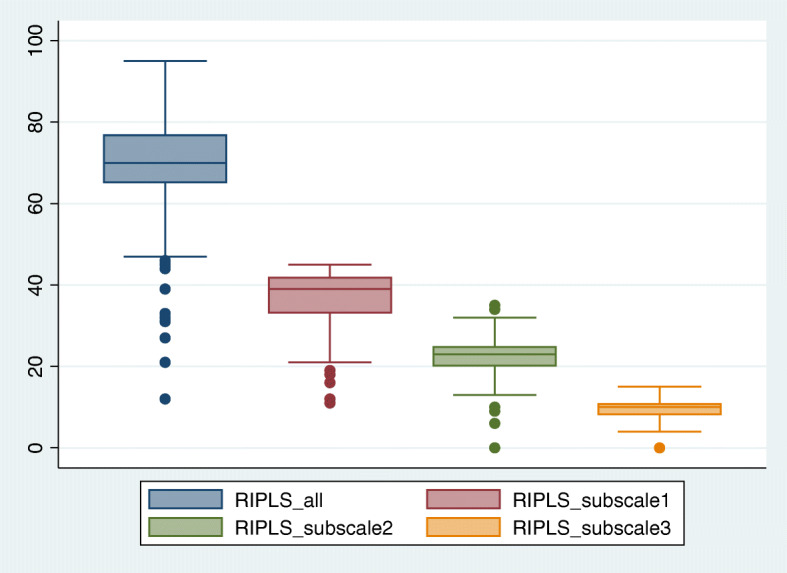
Plot Box for the mean and SD for the overall score of Readiness for Inter-professional Learning Scale (RIPLS) scale and for each subscale: Teamwork and Collaboration (subscale 1), Professional Identity (subscale 2), and Roles and Responsibility (subscale 3)

The overall mean score for the RIPLS was 86.8 (SD ±11.6). The mean score for each statement in the RIPLS is presented in Supplement Table 1. The highest mean score, 4.3 (SD ± 0.9) was obtained for the statement “Shared learning with other healthcare students will increase my ability to understand clinical problems.” Regarding the domain comparison, the mean score for the Teamwork and Collaboration subscale, was 36.6 (SD ± 7.4), the Professional Identity subscale 22.4 (SD ± 4.4), and the Roles and Responsibility subscale 9.7(SD ± 2.3). Figure 1 displays the plot box for the mean and SD for the overall score and for each subscale.

The results of the mean score for the different specialties indicate differences in opinion about the Roles and Responsibility subscale (Table 2). There were statistically significant differences between programs as determined by one way ANOVA (F(6,226) = 3.7, *P*-value = 0.001). The Tukey post-hoc test indicated a statistically significant difference comparing the Occupational Therapy and the Respiratory Therapy programs, the Cardiovascular Technology and Emergency Medical Services programs, and the Anesthesia Technology and Respiratory Therapy programs. There were no statistical significant differences between the other programs.

The independent t-test, done to determine if there is a difference in the RIPLS mean score between male and female students, indicated no difference between the overall RIPLS and the subscales (Table 2). There was no statistically significant difference between the RIPLS mean score overall or the subscales between junior and senior students (Table 3).

### The interdisciplinary education perception scale (IEPS)

**Fig. 2:**
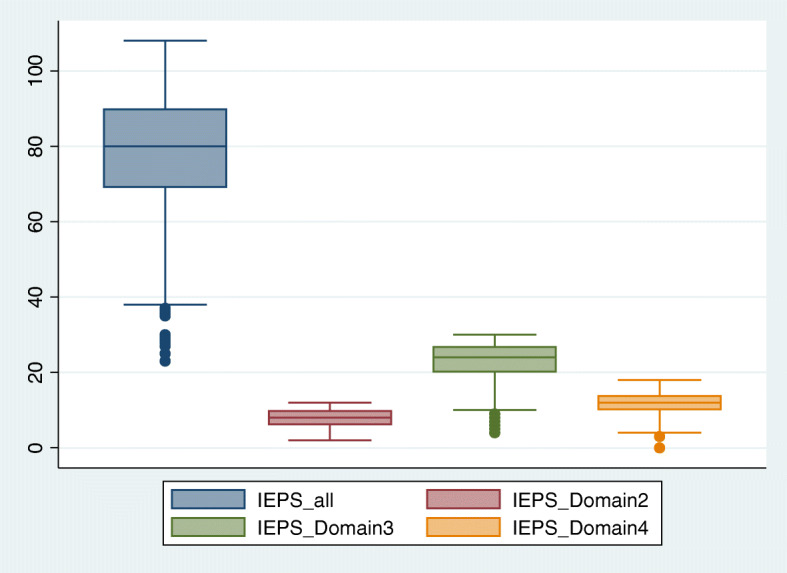
Plot Box for the mean and SD for the overall score of Interdisciplinary Education Perception Scale and for each subscale: Competence and Autonomy (Domain 1) Perceived need for Cooperation (Domain 2), Perception for Actual Cooperation (Domain 3), and Understanding Others’ Value (Domain 4)

The overall mean score for the sample was 77.7 (SD 16.8). Figure 2 displays the distribution of the mean score as well as the mean score for each domain. The mean score for each statement in the IEPS questionnaire is available in Supplement Table 2. Table 1 displays the IEPS domain scores for the various programs. The students in the various programs differed in terms of the Competence and Autonomy domain, perception for Actual Cooperation domain, and Understanding others’ Value domain (Table 1). Based on the results of the one-way ANOVA, there was a significant difference in the mean score for the IEPS domain between the students of the different programs, (F(6,226) = 3.25, *p*-value =0.004) for the Competence and Autonomy domain, (F(6,226) =2.92, *p*-value = 0.009) and the Perception of the Actual Cooperation domain. The Tukey post-hoc test indicated that the score was significantly higher for the Competency and Autonomy domain, comparing the Cardiovascular Technology and Respiratory Therapy programs (*p*-value = 0.03) and the Cardiovascular Technology and Anesthesia Technology programs. The Tukey post-hoc test indicated a significantly higher score for the Occupational Therapy and Respiratory Therapy programs (*p* -value = 0.04) for the Actual Cooperation domain.

**Table 1:** Mean score among different specialty for each subscale of the Readiness for Interprofessional Learning Scale (RIPLS) and for each domain in the Interdisciplinary Education Perception Scale (IEPS)

Subscale	Overall Mean (SD)	Respiratory Therapy	Emergency medical services	Occupational therapy	Radiology Science	Clinical Lab Studies	Anesthesia Technology	Cardiovascular Technology	F-ratio	P
Readiness for Interprofessional Learning Scale (RIPLS)										
**Teamwork and Collaboration**	36.6 (7.4)	36.7 (7.0)	36.8 (8.9)	37.4 (5.8)	37 (8.1)	35 (4.5)	33.1 (9.2)	38.9 (4.6)	F(6,226) = 1.69	0.12
**Professional Identity**	22.4 (4.44)	22.1 (4.5)	23.3 (5.5)	22.8 (2.9)	32.1 (4.3)	20.4 (3.1)	21.5 (5.3)	22.8 (3.4)	F(6,226) = 1.29	0.26
**Roles And Responsibility**	9.7 (2.3)	10.2 (2.1)	9.7 (2.1)	8.6 (1.9)	10.5 (2.6)	8.5 (1.7)	10.2 (2.8)	9.6 (1.7)	F(6,226) = 3.7	0.001
Interdisciplinary Education Perception Scale (IEPS)										
**Competency and Autonomy**	34.9 (8.1)	33 (7.6)	34.6 (9.6)	37 (5.3)	35.7 (7.5)	33.4 (9)	32 (8.6)	39 (7.8)	F(6,226) = 3.25	0.004
**Perceived Need for Cooperation**	7.9 (2.2)	8.3 (1.9)	8.1 (2.3)	7.8 (2.3)	7.5 (2.5)	7.6 (1.4)	7.2 (2.2)	8.6 (2.4)	F(6,226) = 1.45	0.19
**Perception for Actual Cooperation**	22.7 (5.6)	20.8 (5.6)	23.7 (5.9)	24.4 (3.9)	22.2 (6.1)	22 (5.6)	21 (6.2)	24.6 (4.7)	F(6,226) = 2.92	0.009
**Understanding Others’ Value**	12 (3.5)	11.6 (2.6)	12.4 (4.4)	11.8 (2.6)	12.7 (4.1)	12.6 (3)	10.6 (3.9)	13.5 (2.8)	F(6,226) = 2.16	0.05

Table 2 presents the independent t-test results for the difference in the mean IEPS score for male and female students, with no statistically significant difference between the overall and subdomains between the two groups. For the mean difference in the score for junior and senior students, the junior students had a lower score in the Competence and Autonomy domain (7.7 ± 2 score) compared to the senior students (33 ± 7 score, *p*-value = 0.02), which was statistically significant (Table 3).

**Table 2:** Mean score for male and female students for each subscale of the RIPLS and each domain in the IEPS

Scale	Gender		***p***-value
	Male (mean ± SD)	Female (mean ± SD)	
**Readiness for Interprofessional Learning Scale (RIPLS**)			
Overall	68 (13.1)	69.4 (9.5)	0.48
Teamwork and collaboration	36 (8.0)	37.3 (6.6)	0.19
Professional identity	22.4 (5.0)	22.5 (3.6)	0.79
Roles and responsibility	9.9 (2.4)	9.5 (2.0)	0.22
**Interdisciplinary Education Perception Scale (IEPS)**			
Over all	78.6 (17.3)	76.5 (16.1)	0.33
Competency and Autonomy	35.7 (8.6)	34.1 (7.5)	1.15
Perceived need for cooperation	7.9 (2.3)	7.9 (2.1)	0.98
Perception for actual cooperation	22.9 (5.4)	20.4 (5.8)	0.45
Understanding others’ value	12.1 (3.5)	12.1 (3.4)	0.87

**Table 3:** Mean score for junior and senior students for each subscale of the Readiness for Inter-professional Learning Scale (RIPLS) and for each domain in the Interdisciplinary Education Perception Scale (IEPS)

Subscale	Year of study		***P*** value
	Junior (mean ± SD)	Senior (mean ± SD)	
**Readiness for Interprofessional Learning Scale (RIPLS)**			
Overall	70.1 (10.1)	67.6 (12.9)	0.09
Teamwork and collaboration	37.3 (6.9)	35.9 (7.9)	0.17
Professional Identity	22.8 (4.1)	22.11 (4.7)	1.17
Roles and Responsibility	9.98 (2.1)	9.51 (2.4)	0.12
**Interdisciplinary Education Perception Scale (IEPS)**			
Over all	79.1 (17.7)	76.3 (15.7)	0.21
Competency and Autonomy	36.1 (8.4)	33.8 (7.8)	0.02
Perceived need for Cooperation	7.75 (2.2)	8.1 (2.2)	0.2
Perception of Actual Cooperation	22.9 (6.1)	22.4 (5.1)	0.48
Understanding Others’ Value	12.18 (3.8)	11.9 (3.1)	0.62

## Discussion

### Key findings

In the current study, we aimed to identify the perceptions and readiness of undergraduate CAMS students in terms of IPE. The results indicate that the students from all the programs have positive attitudes toward IPE. The students from the different programs in the college enroll in Research Methodology I and II as mandatory courses, facilitating interaction and working as a whole team during the Research Methodology’s lectures and assignments.

The majority of the students in the different programs recognizes and has been prepared to receive IPE, as an important element in creating collaborative teamwork. The difference in the degree of the students’ preparedness toward IPE may reflect different interprofessional practices, implemented within each program.

The CAMS programs contain fieldwork courses as an integral part in all the programs offered at the college at undergraduate level. The main learning outcome of the fieldwork courses is to enhance the student’s interpersonal skills and encourage collaboration with the community. To illustrate, practicum courses are listed as a mandatory course in all curricula. The Respiratory Therapy program have five practicum courses. Students in these courses are required to have clinical rotations at the university’s hospital. In these clinical rotations, students have the chance to communicate, interact and work as a team with each other and with hospital staff in different levels. The Emergency Medical Services program students are exposed to the Respiratory Therapy in the Emergency Units, and the Radiologic Science students have daily interaction with the Respiratory Therapy students in the Intensive Care Units. In addition, the Occupational Therapy students work with Respiratory Therapy students in the Cardiopulmonary Rehabilitation units. This work dynamic improves patient care. Students from these specialties are having the opportunity to discuss, manage, and treat a patient as a team and with the different hospital specialties.

### Comparison to other studies

In the current study, the higher mean scores of the RIPLS, in the Teamwork and Collaboration subscales, are consistent with literature [12]. Support is also available from studies done with medical students appreciating shared learning and teamwork to provide patients with high-quality patient care [13, 14]. In addition, a study conducted with Medical and Dental students in Princess Nourah bint Abdulrahman University, reported that students are aware that collaboration with other specialties will improve their service to patients and the community [15]. Regarding the professional identity subscale, all students agreed with the statements “Shared learning before qualification will help me become a better team worker.” “Shared learning will help to clarify the nature of patient problems” and “Shared learning with other healthcare students will help me to communicate better with patients and other professionals.” These statements correlate with a good understanding of teamwork and a positive conviction of shared learning. As all the participants in the current study were from the same college, studying together in the pre-professional years according to the university policies, their professional identity increased from the first year of study [15]. However, the highest-ranking statement was “The function of nurses and therapists is mainly to provide support for doctors” in the role and responsibility with other applied medical sciences specialties. This subscale item suggests that students have a negative view in their role and responsibility, which may be the result of a different curriculum structure and the short period of specialized study. Students need a more focused explanation of their role in a healthcare institution, in addition to their independent responsibility of specializing in diverse majors [2].

The findings from this study also indicated that senior students had higher IEPS scores compared to junior students. This could be explained with the senior students having more experience and exposure to the healthcare environment during the practicum course. Reeves et al. also reported a difference in attitude and perception between senior and junior students [16]. It is noteworthy that there were no gender differences in the IEPS score, indicating that there is no undue importance between male and female in the IPL. This result provides an important perspective that should be considered in planning and recruitment policies in hospitals.

### Strength and limitations

A strength of the current study is exploring the perceptions and readiness of undergraduate students towards IPE in health-related professions, not reported in literature for the Respiratory Therapy and Radiology Program. A limitation of the study is that the nature of the study design may have affected the study outcome due to the possibility of selection bias and the inability to measure interpersonal confounders, such as previous experience or receiving other courses. In addition, the generalizability of the outcome may be limited as it was conducted in one university. Nevertheless, the study is one of a few studies contributing to the deficit in literature regarding the perceptions of students oh IPE from populations, other than medical students. Only a few studies are available in literature regarding the perceptions and readiness of undergraduate students toward IPE in Saudi Arabia’s universities.

## Conclusion

The current study found that the College of Applied Medical Sciences’ students demonstrated readiness for IPE as an important element in creating collaborative teamwork in their programs. However, there is a difference in the readiness of the students in some programs in CAMS, supporting an early exposure of the students from those programs to IPE in their curricula. The current study also indicated that senior students have more positive perceptions and a higher level of readiness for IPL than the junior students. This support the recommendation for the early incorporation of IPE in the pre-professional years to enhance collaboration in management and patient care. Additional studies should implement triangulation methods and conduct longitudinal studies, a strategy that will advance the inter-professional education and interdisciplinary collaboration.

## Supplementary Information

**Additional file 1: Supplement Table 1.** Readiness for Interprofessional Learning Scale (RIPLS), item-level analysis. **Supplement Table 2.** Interdisciplinary Education Perception Scale (IEPS) item-level analysis.

## Data Availability

The datasets used and analyzed during the current study are available from the corresponding author on a reasonable request.

## Abbreviations

IPE: Interprofessional education
IOM: The Institute of Medicine
IEPS: Interdisciplinary Education Perception Scale
RIPLS: Readiness for Interprofessional Learning Scale
IPL: Interprofessional Learning
CAMS: College of Applied Medical Science
