# Supplementary material for: Students’ readiness for and perception of Interprofessional learning: a cross-sectional study
Source: BMC Med Educ. 2020 Oct 29;20:390. doi: 10.1186/s12909-020-02325-9 (PMC7597065; doi:10.1186/s12909-020-02325-9)
Supplement: Supplementary file 1 — Additional file 1: Supplement Table 1. Readiness for Interprofessional Learning Scale (RIPLS), item-level analysis. Supplement Table 2. Interdisciplinary Education Perception Scale (IEPS) item-level analysis. [file 12909_2020_2325_MOESM1_ESM.docx]

**Supplement tables:**

Supplement Table 1: Readiness for Interprofessional Learning Scale (RIPLS), item-level analysis

| number | Item statement | Mean score | Standard deviation |
| --- | --- | --- | --- |
| 1 | Learning with other students will help me become a more effective member of a healthcare team. | 4.1 | 1.1 |
| 2 | Patients would ultimately benefit if healthcare students worked together to solve patient problems. | 4.2 | 1.0 |
| 3 | Shared learning with other healthcare students will increase my ability to understand clinical problems. | 4.3 | 0.9 |
| 4 | Learning with healthcare students before qualification would improve relationships after qualification. | 4.0 | 1.1 |
| 5 | Communication skills should be learned with other healthcare students. | 4.1 | 1.0 |
| 6 | Shared learning will help me to think positively about other professionals | 4.0 | 0.9 |
| 7 | For small-group learning to work, students need to trust and respect each other. | 3.9 | 0.9 |
| 8 | Teamwork skills are essential for all healthcare students to learn. | 4.0 | 1.0 |
| 9 | Shared learning will help me to understand my own limitations. | 4.2 | 1.0 |
| 10 | I do not want to waste my time learning with other healthcare students. | 2.1 | 1.2 |
| 11 | It is not necessary for undergraduate healthcare students to learn together. | 2.2 | 1.2 |
| 12 | Clinical problem-solving skills can only be learned with students from my own department. | 2.6 | 1.1 |
| 13 | Shared learning with other healthcare students will help me to communicate better with patients and other professionals. | 3.9 | 1.1 |
| 14 | I would welcome the opportunity to work on small-group projects with other healthcare students. | 3.8 | 1.0 |
| 15 | Shared learning will help to clarify the nature of patient problems. | 3.9 | 1.1 |
| 16 | Shared learning before qualification will help me become a better team worker. | 4.0 | 0.9 |
| 17 | The function of nurses and therapists is mainly to provide support for doctors. | 4.0 | 1.0 |
| 18 | I am not sure what my professional role will be. | 2.2 | 1.2 |
| 19 | I have to acquire much more knowledge and skills than other healthcare students | 3.5 | 1.1 |
|  | **overall** | **86.8** | **11.6** |

Supplement Table 2: Interdisciplinary Education Perception Scale (IEPS) item-level analysis

| **number** | **Item statement** | **Mean score** | **Standard deviation** |
| --- | --- | --- | --- |
| 1 | Individuals in my profession are well trained. | 4.6 | 1.2 |
| 2 | Patients would ultimately benefit if healthcare students worked together to solve patient problems. | 4.5 | 1.2 |
| 3 | Individuals in my profession are able to work closely with individuals in other professions. | 4.6 | 1.1 |
| 4 | Individuals in my profession demonstrate a great deal of autonomy. | 4.3 | 1.3 |
| 5 | Individuals in my profession are very positive about their goals and objectives. | 4.5 | 1.3 |
| 6 | Individuals in my profession need to cooperate with other professions. | 4.6 | 1.3 |
| 7 | Individuals in my profession are very positive about their contributions and accomplishments. | 4.6 | 1.2 |
| 8 | Individuals in my profession must depend upon the work of people in other professions. | 3.3 | 1.5 |
| 9 | individuals in other professions think highly of my profession | 3.8 | 1.3 |
| 10 | Individuals in my profession trust each other’s professional judgment. | 4.3 | 1.2 |
| 11 | Individuals in my profession have a higher  status than individuals in other professions | 3.9 | 1.2 |
| 12 | Individuals in my profession make every effort to understand the capabilities and contributions of other professions. | 4.2 | 1.2 |
| 13 | Individuals in my profession are extremely competent. | 4.5 | 1.2 |
| 14 | Individuals in my profession are willing to  share information and resources with other professionals. | 4.7 | 1.2 |
| 15 | Individuals in my profession have good relations with people in other professions. | 4.7 | 1.2 |
| 16 | Individuals in my profession think highly of other related professions. | 4.2 | 1.2 |
| 17 | Individuals in my profession work well with each other. | 4.7 | 1.3 |
| 18 | Individuals in other professions often seek the advice of people in my profession. | 4.1 | 1.3 |
|  | overall | 77.7 | 16.8 |
